# Cooling the Conversation: Discourse About Menthol and Flavored Tobacco Restrictions on TikTok

**DOI:** 10.1080/10826084.2026.2621980

**Published:** 2026-02-01

**Authors:** Charlotte McCormick, Nathan A. Silver, Susana Rodriguez Gongora, George D H Pearson, Page D. Dobbs

**Affiliations:** a Department of Health, Human Performance and Recreation, University of Arkansas, Fayetteville, Arkansas, USA; b Center for Public Health and Technology, University of Arkansas, Fayetteville, AR, USA; c Schroeder Institute at Truth Initiative; d Department of Health Behavior and Health Education, Fay W. Boozman College of Public Health, University of Arkansas for Medical Sciences, Fayetteville, Arkansas, USA

**Keywords:** menthol ban, flavored tobacco ban, TikTok, social media, tobacco control, tobacco regulatory science

## Abstract

**Introduction:**

TikTok promotes methods that circumvent commerce laws and advances disinformation about tobacco policies. Currently, flavor-related policies are contentious tobacco control issues. This paper examines flavor bans content on TikTok.

**Methods:**

TikTok videos (*n* = 1,139) and metadata posted between September 2018 and September 2023 were scraped using a TikTok application programming interface (API) and popular hashtags related to flavor ban content (i.e., #menthol [135.3 million]), #mentholban [11.7 million], #nonmenthol [25K]), #flavorban [3.9 million]. Data were classified for relevance to menthol bans and emergent codes by two trained coders.

**Results:**

Flavor-ban-related videos (*n* = 609) displayed cigarettes (51.6%), e-cigarettes (23.9%), and nicotine pouches (2.0%). Overall, 9.5% of the videos were filmed in retail settings such as vape shops (6.4%) and gas stations (2.6%). Policy circumvention methods appeared in videos promoting flavor capsule injectors (13.3%), discussing non-menthol cigarettes (4.1%), and displaying other circumvention methods (e.g., flavor cards; 12.6%). Videos that depicted methods of circumventing tobacco control policies received 66.6% of all views and 31.4% of all likes. Influencers discussed menthol bans in 226 (37%) videos; 58.4% were against the ban, 9.3% in support, and 32.3% were neutral. Among influencer videos, 17.7% focused on racial implications, with 55% opposing the ban and 32.5% supported it. These discussions received 11.2% of all views and 25.3% of all likes.

**Conclusions:**

Although videos aimed at policy discussions and political mobilization are noteworthy, those promoting circumvention strategies were viewed more often. TikTok’s role for underregulated marketing and sales of tobacco products and accessories is a pressing tobacco regulatory issue.

## Introduction

Policy interventions worldwide seek to restrict flavored tobacco and nicotine products in order to reduce youth initiation and aid cessation (Erinoso et al., 2020). Although most flavored combustible products were removed from the US market in 2009, menthol as a characterizing flavor remains, reducing barriers to initiation and making cessation harder (Mills et al., 2025; Smiley & Shin, 2024). Like other flavors, menthol reduces the harshness of smoking, making it easier for first-time users (Carstens & Carstens, 2022). Additionally, menthol smokers report higher dependence and lower quit success than non-menthol smokers (Wickham, 2020), providing strong evidence in support of comprehensive restrictions (Cadham et al., 2020). In the absence of a national menthol restriction law, state and local laws have been enacted across the U.S. (Donovan et al., 2021). However, these laws are hampered by loopholes and enforcement challenges that merit further investigation.

The emergence of illicit markets providing access to banned products is an often-highlighted limitation of flavor restrictions (Brown et al., 2023; Freitas-Lemos et al., 2023; Yang et al., 2024). Social media platforms have emerged as a vector for information about policy circumvention methods (Kostygina et al., 2022; Silver et al., 2022) with TikTok providing an illicit market for product promotion, demonstration, and distribution (Dobbs et al., 2025; Donaldson et al., 2026; Pearson et al., 2025).

Most research examining the role of social media on menthol and flavor restrictions has predominantly focused on discourse related to the efficacy and appropriateness of a total ban (Allem et al., 2023; Zhou et al., 2023). This research identifies key arguments disseminated online that may drive opposition to such policy, such as discourse suggesting a menthol restriction will lead to racial discrimination (given preference for menthol cigarettes among African American/Black people who smoke) and that illicit markets will emerge in response to a ban (Allem et al., 2023; Dobbs et al., 2026; Feng et al., 2024; Silver et al., 2025; Zhou et al., 2023). Social media has the ability to focus public attention on specific issues (Gilardi et al., 2022; Wu & Coleman, 2009) and emphasize specific attributes of those issues over others (i.e., framing) (Entman, 1993) to drive public sentiment. Additionally, media systems, like social media, can provide insight to information deficient environments, such as those that follow a rapidly evolving policy landscape (Ball-Rokeach & DeFleur, 1976; Kim & Jung, 2017). Thus, social media discourse, within a particular context and framework can provide important insights into community responses to policies. Although TikTok is a global platform, the current study interpreted data within a US policy framework. Therefore, the purpose of this study was to examine menthol and flavor-related content for both US policy-related discourse as well as information about alternative products, circumvention, and other responses in TikTok videos posted between 2018 and 2023.

## Methods

### Data collection

September 2023, 1,139 public TikTok videos and metadata were collected using a TikTok application programming interface (API; https://console.apify.com/). We identified hashtags using a snowball procedure that considered prior literature (Feng et al., 2024), manually explored hashtags, and prioritized those specific to menthol restriction policies. This included five rounds of hashtag review, leading to the removal of #crushballs because of the limited focus of posts. The final hashtags included: #menthol (135.3 million views), #mentholban (11.7 million views), #nonmenthol (25,000 views), and #flavorban (3.9 million views). Eligible videos were publicly posted in the past five years (September 12, 2018–September 12, 2023), a period during which multiple state-level menthol restriction laws went into effect and the US Food and Drug Administration (FDA) announced a proposed restriction on combustible menthol-flavored tobacco products. Data were not limited by language because analysis considered visual depictions. After removing irrelevant data (e.g., menthol discussions not related to tobacco), the analytic sample included 609 relevant videos. All procedures were exempt from review by the University of Arkansas Institutional Review Board.

### Coding and analysis

The full analytic sample was coded by authors CM and SRG with discrepancies resolved by PDD. An initial 100 videos were coded to establish conceptual clarity and finalize the codebook, after which 200 videos were coded weekly followed by additional discussions to address discrepancies. Interrater reliability for the final week of coding exceeded convention (0.7; Cohen, 1960). See Table 1 for complete coding criteria, examples, and reliability metrics. Descriptive analysis of metadata including median views and likes between coded content were used to characterize reach and engagement.

## Results

**Figure 1.**
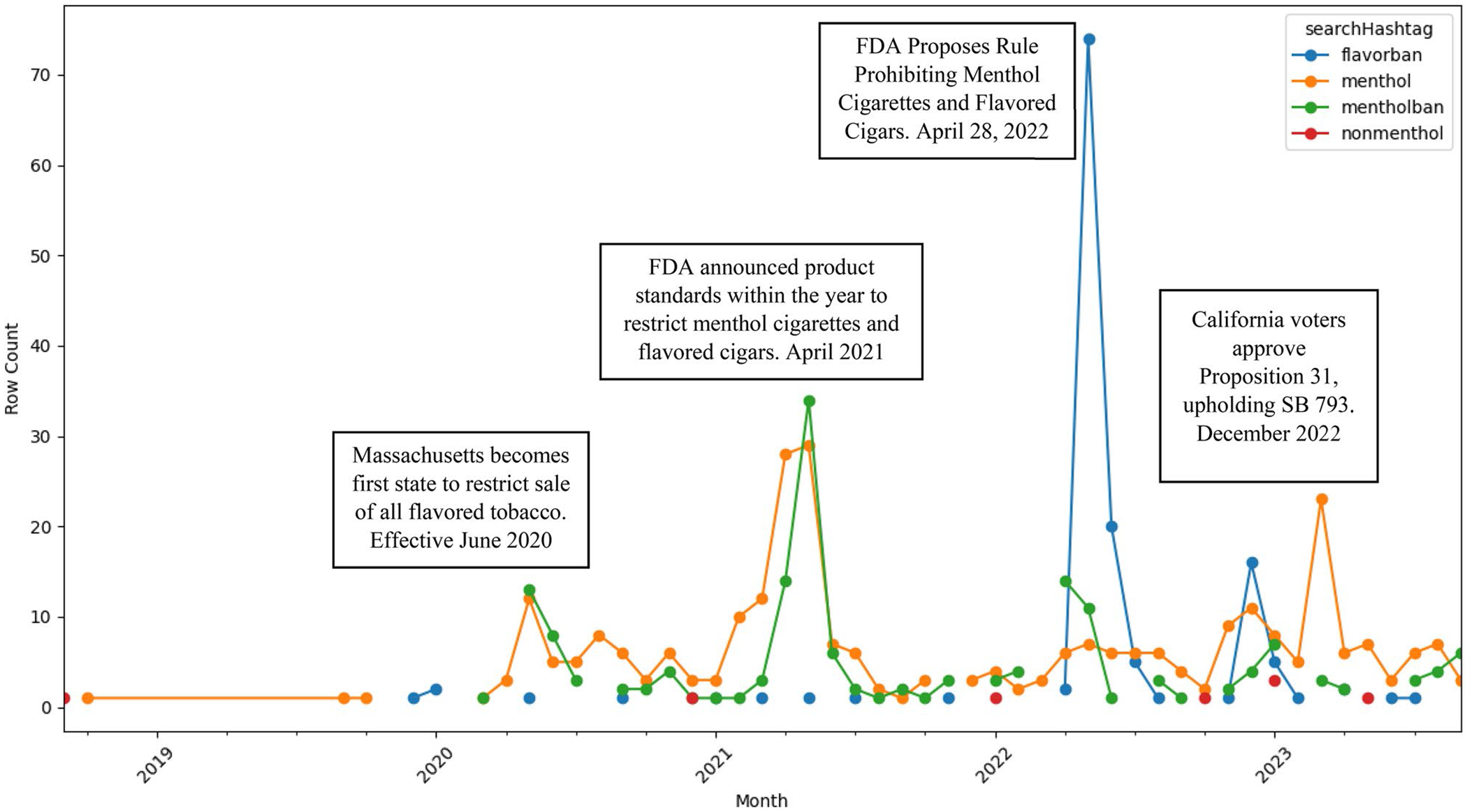
Monthly count of posts included searched hashtags about flavor restriction laws posted on TikTok from 2018 to 2023.

The 609 videos determined relevant to tobacco flavor restrictions were viewed 44,798,241 times and liked 1,564,517 times. Spikes in posts using #menthol and #menthol ban occurred around the time that state (Massachusetts, June 2020; California, December 2022) policies went into effect and when the FDA announced a product standard restricting menthol cigarettes and flavored cigars would be released within the year. Peak discourse occurred with the hashtag #flavorban after FDA released the proposed rule on May 04, 2022. See Figure 1 for monthly TikTok videos posted. Posting authors were liked and followed by a median of 40,000 (range = 0–80,400,000) and 2,318 (range = 7-1,600,000) users, respectively. Among our sample, 51.6% (*n* = 315) displayed cigarettes and 23.9% (*n* = 146) displayed e-cigarettes; 2.0% displayed nicotine pouch products. Few videos were visibly filmed in a vape shop (6.4%) or gas station (2.6%); these videos displayed retailers explaining the law. See Table 2.

### Social media influencers disseminating information about flavor restriction laws

Overall, 37.0% (*n* = 226) of the videos displayed people discussing the laws. Posting authors of these posts had a median of 4,731 followers per video and had been liked a median of 107,200 times. Further, 75.2% (*n* = 170) of videos that displayed someone discussing the law were posted by social media influencers, having at least 1,000 followers (Vassey et al., 2023) (range = 1,042-1,600,000). Influencer videos received 11.2% (5,019,199) of all views and 25.3% (395,446) of all likes.

### Methods of circumventing flavor restriction laws

Methods of circumventing included the display or advertisements of non-menthol menthol cigarettes, promotion of flavor injection devices, and other circumventing. Overall, 4.1% (*n* = 25) of the videos displayed non-menthol cigarettes advertised by color instead of flavor, and 13.9% displayed (*n* = 85) injection devices that insert crush balls into the filter of a cigarette. Another 12.6% (*n* = 77) discussed methods of circumventing flavor restriction laws, such as using menthol cards inside cigarette packages to absorb the flavor and providing tips on where to buy flavored tobacco products illegally. Most circumvention videos involved combustible cigarettes rather than e-cigarettes. Circumvention methods-focused videos received 66.6% (29,848,171) of all views and 31.4% (491,654) of all likes.

## Discussion

Circumvention strategies for flavor restriction laws were promoted on TikTok during a timeframe when Massachusetts and California enacted menthol flavor restriction laws (Campaign for Tobacco Free Kids, 2024). These findings raise public health concerns given TikTok’s popularity among adolescents and young adults (Pew Research Center, 2024). Unlike menthol-ban-related discussions on Twitter and Reddit, which often discussed the policies themselves (Allem et al., 2023; Silver et al., 2025; Zhou et al., 2023), the TikTok videos that generated the most views in our sample focused on policy circumvention, specifically flavor additives that violate California’s flavor restriction law (§ 104559.5) that includes flavor enhancers. Public perception of the effectiveness of these policies may be diminished if the public perceives circumvention to be easy. Alternatively, awareness of this information may impact policy agenda that seek to close loopholes, consistent with the agenda setting theory (Kostygina et al., 2022).

TikTok appears uniquely suited to disseminate products and demonstrations of products that circumvent tobacco flavor restriction laws, such as crush balls and flavor cards (Chaiton et al., 2021; Dobbs et al., 2025), due to the visual nature of the platform. This is not to say policy circumvention discourse is isolated to TikTok, as prior research documented similar discussions of circumvention strategies on Instagram and Reddit for flavored e-cigarettes (Kostygina et al., 2022; Silver et al., 2022). However, prevalence of such content on TikTok, a platform dominated by young audiences, affirms prior research identifying the need to address illicit markets targeted to youth and young adults online (Dobbs et al., 2025; Pearson et al., 2025). Similar to other platforms, TikTok is a self-regulated entity and balances platform algorithms and engagement strategies with restriction guidelines (Watti et al., 2023). TikTok’s community guidelines prohibit the promotion and use of “regulated goods and services.” However, “products like alcohol” are allowed with some restrictions (TikTok, 2025). Therefore, it is unclear whether demonstrations or use of menthol products or circumvention strategies violate these self-regulated policies.

Although the FDA proposed a product standard to prohibit the sale of menthol cigarettes on May 04, 2022 (21 CFR Part 1162), implementation timelines and political feasibility remain uncertain. Therefore, state and local jurisdictions are likely to continue leading efforts to restrict flavored tobacco products (Kruse et al., 2025). State and local governments seeking to restrict flavored tobacco products should be aware of flavored capsules used to circumvent these laws (Dobbs et al., 2025). Laws, such as California’s, explicitly restrict flavor enhancers, making it illegal to sell these products in retail locations in the state. However, loopholes remain as these products are sold online. In states where the law is less clear about flavor enhancers (i.e., Massachusetts), it is less likely that courts would restrict these products under their code.

### Limitations

The current sample was limited in scope by the hashtags employed and the time frame from where data were collected. Only publicly available videos were annotated, meaning those whose accounts are private were not viewed or reflected in this data. Therefore, content from private accounts or less discoverable videos may be different in tone, audience, or displayed agenda. Lastly, it is possible that videos were removed (either by the uploader or moderated by TikTok) before data collection.

## Conclusions

Video-based platforms expand communication and create enforcement challenges across jurisdictions. Retailers and influencers can promote products that circumvent tobacco flavor restriction laws. To counter this, public health agencies should leverage communication campaigns on TikTok that disseminate evidence-based prevention messages. Further, state and federal regulatory agencies should work closely with platforms to detect illegal activity within jurisdictions that restrict flavored tobacco and enforce tobacco advertisement restrictions that align with the World Health Organizations’ Framework Convention on Tobacco Control Article 13. Lastly, closing regulatory loopholes, such as banning online flavored accessory sales and enforcing tobacco delivery laws, could help ensure such laws are implemented as intended.

## Figures and Tables

**Table 1.** Codebook of TikTok videos about menthol and flavor restriction policies (*n* = 609).

Code (subcodes)	Operational Definition	Kappa
Product Type		
Cigarettes	Displayed a cigarette	0.78
Non-Menthol Menthol Cigarettes	Displayed or discussed non-menthol menthol cigarettes.	1.0
E-cigarettes	Displayed e-cigarettes	0.85
Nicotine pouches	Displayed nicotine pouches	1.0
Flavor Injector	Demonstrates how to use flavor injector/crush balls	0.95
Race	Discussion includes a reference to race or ethnicity.	0.80
Rights	Discussion includes someone that talks about individual freedoms or rights.	1.0
People Discussing Law Influencer Sentiment	Displays a person discussing the law.	0.91
Positive	Discussion in the video about the flavor restriction law is Positive, supports the law.	0.80
Negative	Discussion in the video about the flavor restriction law is negative, includes venting about the law.	0.79
Neutral	Discussion in the video about the flavor restriction law is neutral, only mentions it but doesn’t express support or opposition.	0.79
Location		
Vape shop	Video is filmed in a Retail - Vape shop – You can see that all products sold are vape products	1.0
Gas station	Video is filmed in a Retail – Gas Station – You can see that the retail space is a gas station	1.0
Circumvent	Discusses a way to circumvent the law not already covered by the non-menthol menthol cigarettes or flavor injector device.	0.87

**Table 2.** Classification of TikTok videos about menthol and flavor restriction policies (*n* = 609).

Code (subcodes)	N (%)
Product Type	
Cigarettes	315 (51.6)
Non-Menthol Menthol Cigarettes	25 (4.1)
E-cigarettes	146 (23.9)
Nicotine pouches	12 (2.0)
Flavor Injector	85 (13.9)
Race	44 (7.2)
Rights	13 (2.1)
People Discussing Law	226 (37)
Influencer Sentiment	
Positive	21 (3.4)
Negative	132 (21.6)
Neutral	73 (12.0)
Location	
Vape shop	39 (6.4)
Gas station	16 (2.6)
Circumvent	77 (12.6)

## Data Availability

Data will be made available upon request.
